# A Case of Closed Pilon Fracture Resulting in Soft-Tissue Necrosis and Treated with Reverse Sural Artery Flap and Circular External Fixation

**DOI:** 10.1155/2023/9222479

**Published:** 2023-08-09

**Authors:** Ryu Igaki, Tomohiro Yasuda, Yuki Samejima, Yuko Irie, Yuto Murakami, Taisuke Yoneya, Shinsuke Takagi, Keikichi Kawasaki, Koji Kanzaki

**Affiliations:** ^1^Department of Orthopaedic Surgery, Showa University Fujigaoka Hospital, Yokohama, Japan; ^2^Department of Orthopaedic Surgery, Yokohama Shintoshi Neurosurgical Hospital, Yokohama, Japan; ^3^Department of Plastic and Reconstructive Surgery, Showa University School of Medicine, Tokyo, Japan; ^4^Department of Orthopaedic Surgery, Showa University Northern Yokohama Hospital, Yokohama, Japan

## Abstract

Tibial pilon fractures are difficult to treat due to articular comminution and soft-tissue injuries caused by high-energy trauma. Open reduction and internal fixation is a commonly used method of treatment. However, it has a high risk of infection and soft-tissue complications due to the extensive detachment of soft tissue. We report on a case with a tibial pilon fracture and soft-tissue necrosis that we treated using limited internal fixation combined with a circular external fixator (LIFCEF) and reverse sural artery flap (RSAF) as part of an orthoplastic approach within the orthopedic surgery department alone, which obtained good results. A 51-year-old man was injured in a motorcycle accident and transported to a nearby hospital. X-rays at the time of injury showed tibial pilon fractures (AO Foundation/Orthopedic Trauma Association 43c3.3, Ruedi–Allgower: Type III). Soft-tissue necrosis with blisters on the medial side of the lower leg (AO soft-tissue classification: IC3-MT1-NV1) was observed. In addition, the patient was referred to our hospital on day 10 of the injury. LIFCEF was chosen for treating the fracture because plate fixation was accompanied by the risk of plate exposure, soft-tissue complications, and an increased skin defect area, and RSAF was chosen to reconstruct the soft tissue defect. Four years after the surgery, the American Orthopedic Foot and Ankle Score was 92 points. X-ray alignment evaluation showed mLDTA 93° and aADTA 91°. Stage 2 arthrosis was present according to the Takakura ankle osteoarthritis classification, but the patient was able to walk without pain. Tibial pilon fractures are difficult to treat due to articular comminution and soft-tissue injuries caused by high-energy trauma. The timing and choice of treatment are crucial concerning the soft tissue.

## 1. Introduction

Tibial pilon fractures are relatively common, accounting for 5–7% of tibia fractures. These fractures are difficult to treat due to articular comminution and soft-tissue injuries caused by high-energy trauma [1]. In addition, reconstruction of the soft tissue around the ankle joint can be challenging due to the lack of soft tissue [2]. Open reduction and internal fixation (ORIF) is a commonly used method of treatment. However, it has a high risk of infection and soft-tissue complications due to the extensive detachment of soft tissue [3]. To reduce the risk of soft tissue complications, two main treatment approaches have been developed: the staged approach, using external fixation, and the minimally invasive plate osteosynthesis method [4], or limited internal fixation combined with an external fixator (LIFEF), which have become the current mainstream [3]. Another treatment option is joint immobilization, which is rarely used.

We report on a case with a tibial pilon fracture (AO Foundation/Orthopedic Trauma Association [AO/OTA] classification: AO/OTA 43c3.3) and soft-tissue necrosis (AO soft-tissue classification: IC3-MT1-NV1) that we treated using limited internal fixation combined with a circular external fixator (LIFCEF) and reverse sural artery flap (RSAF) as part of an orthoplastic approach within the orthopedic surgery department alone, which obtained good results.

## 2. Case Report

**Figure 1:**
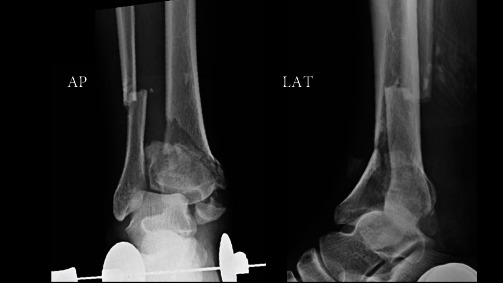
X-rays at the time of injury (after calcaneal traction) show the tibial pilon fractures (AO/OTA 43c3.3, Ruedi–Allogoer: Type III).

A 51-year-old man was injured in a motorcycle accident and transported to a nearby hospital. X-rays at the time of injury showed tibial pilon fractures (AO/OTA 43c3.3, Ruedi–Allgower: Type III; Figure 1).

**Figure 2:**
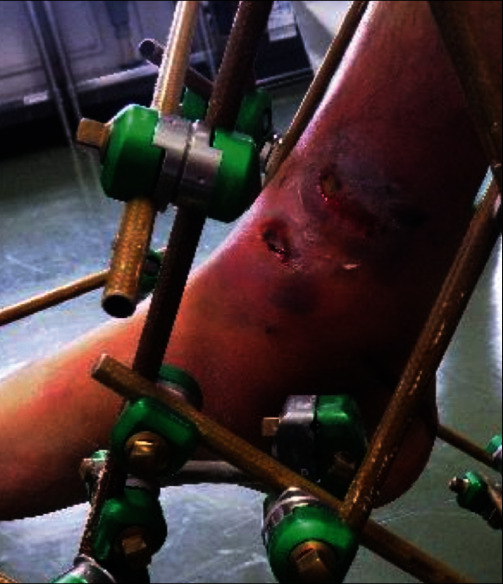
Exterior view after fixation with an external fixator. Skin necrosis and closed fracture with soft tissue necrosis on the medial side of the lower leg (IC3-MT1-NV1).

**Figure 3:**
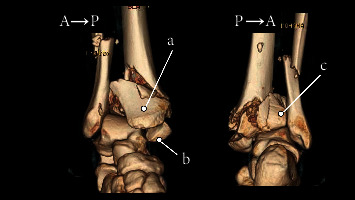
3D CT at the time of the injury. (a) Anterior fragments. (b) Medial fragments. (c) Posterolateral fragment.

**Figure 4:**
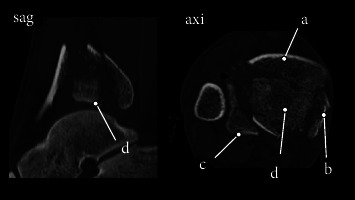
CT at the time of the injury. (a) Anterior fragments. (b) Medial fragments. (c) Posterolateral fragments. (d) Die-punch fragments.

Calcaneal traction was applied on the day of the injury. On day 8 of the injury, temporary spanning external fixation was performed. Soft-tissue necrosis with blisters on the medial side of the lower leg (AO soft-tissue classification: IC3-MT1-NV1) was observed (Figure 2), and the patient was referred to our hospital on day 10 of the injury. A preoperative computed tomography (CT) examination showed that the joint surface was divided into four parts: the anterior fragment (Figures 3(a) and 4(a)), the medial bone fragment (Figures 3(b) and 4(b), the posterolateral bone fragment (Figures 3(c) and 4(c)), and the die-punch fragment (Figure 4(d)). LIFCEF was chosen for treating the fracture because plate fixation was accompanied by the risk of plate exposure, soft-tissue complications, and an increased skin defect area, and RSAF was chosen to reconstruct the soft tissue defect.

**Figure 5:**
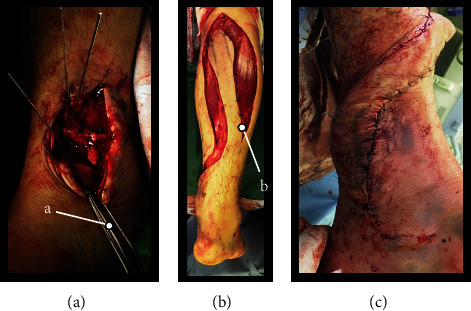
Exterior view during surgery. (A) Restoration of the debrided articular under direct visualization. (B) After removal of the RSAF (10 cm in height and 8 cm in width with 4 cm pedicle with skin). (C) Covered area of skin loss. (a) Temporary fixation using k-wire. (b) Dissected flap.

**Figure 6:**
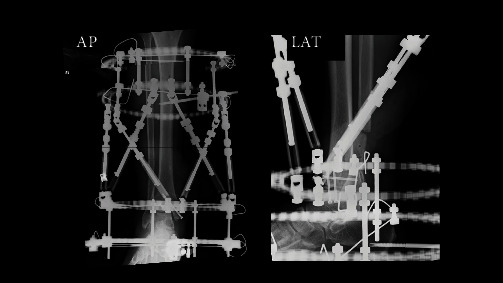
Post-surgery X-ray and circular external fixators. Rings were placed on the tibia at three locations, and a foot ring was placed on the foot at one location.

Surgery was performed on day 12 of the injury. First, we performed debridement of the necrotic soft tissue on the medial side. The area of soft tissue defect was 5 cm × 6 cm. We removed the medial bone fragment from the debrided area and directly visualized the joint surface (Figure 5(a)), which we fixed with a cannulated cancellous screw (CCS). We then fixed the medial bone fragment with two CCSs. Next, the soft tissue defect was reconstructed. Using the Doppler echo, we identified perforators that passed through the superficial sural artery from the peroneal artery about 5 cm proximal to the lateral malleolus as the pivot point for the RSAF. The flap design was enlarged from the area of the damaged defect, measuring approximately 10 cm in length and 8 cm in width (Figure 5(b)). The flap was raised on the proximal side. An incision was made through the skin and subcutaneous tissues. Subsequently, the lesser saphenous vein, medial sural nerve, and superficial sural artery, all of which traverse the subfascial plane, were carefully identified and incorporated into the flap. The fascia was expanded by approximately 3 cm beyond the skin incision, and the skin flap was elevated with an approximately 4 cm pedicle with skin. The flap was dissected subfascially, utilizing the pivot point as the fulcrum, and rotated to cover the defect. It was then sutured into place. External fixation was performed using a circular external fixator. Rings were placed on the tibia at three locations, and a foot ring was placed and fixed on the foot (Figure 6). After the surgery, we allowed full weight bearing and conducted walking training, which started in the third week depending on the pain to prevent skin flap congestion. We administered weekly chlorhexidine dressings to attend to the pin sites. If there is the presence of cutaneous erythema and purulent discharge, oral antibiotics are administered. Bone union was evaluated utilizing CT scans to determine the optimal timing for the removal of the external fixator. The external fixator was removed 20 weeks after the surgery.

**Figure 7:**
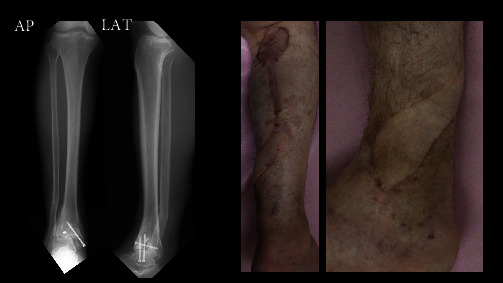
X-ray and the exterior of the RSAF one year after surgery.

**Figure 8:**
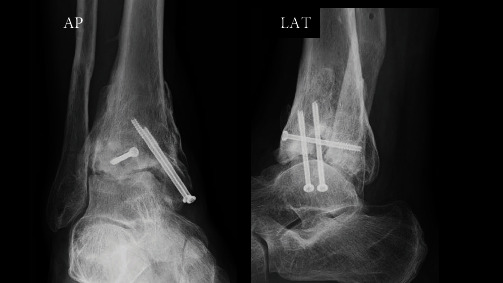
X-ray 4 years after surgery. Degenerative changes were observed in the articular surface (Takakura ankle osteoarthritis classification: Stage 2).

During the one-year follow-up after the surgery, the patient was able to walk without an assistive device and had a range of motion of 15° for dorsiflexion and 30° for plantar flexion at the ankle joint. X-ray evaluation showed stage 2 arthrosis according to the Takakura ankle osteoarthritis classification (Figure 7) [5]. Four years after the surgery, the American Orthopedic Foot and Ankle Score was 92 points. X-ray alignment evaluation showed mLDTA 93° and aADTA 91°. Stage 2 arthrosis was present according to the Takakura ankle osteoarthritis classification, but the patient was able to walk without pain (Figure 8).

## 3. Discussion

Tibial pilon fractures are difficult to treat due to comminuted fractures and soft-tissue injuries [1]. The case involved a closed fracture with soft-tissue necrosis accompanied by comminuted tibial pilon fractures due to articular comminution (AO43c3). Since plate fixation is associated with the risk of plate exposure, we used LIFCEF and RSAF to prevent complications, such as infection and osteomyelitis.

Treatment for tibial pilon fracture is based on the four principles proposed by Ruedi and Allgower, including reconstruction of the fibular length, reconstruction of the joint surface, autologous bone grafting for missing articular surface bone, and buttress plate fixation for the medial side of the fracture. These principles have been reported to yield good results [6]. However, subsequent studies have reported high rates of infection (55%) and soft-tissue necrosis (36%) in patients with high-energy tibial pilon fractures treated with ORIF according to the principles of Ruedi and Allogoer [7]. To reduce the incidence of soft-tissue complications, the use of external fixation has become popular, and the current mainstays of treatment are the staged approach, in which temporary spanning external fixation is followed by internal fixation, or LIFEF [3]. However, in cases such as the present one, with a soft-tissue injury around the joint surface, an orthoplastic approach is necessary. It is widely known that orthoplastic approaches, such as fix and flap, can reduce the incidence of deep infections and improve treatment outcomes in open tibial pilon fractures. However, these approaches are not commonly used for closed tibial pilon fractures [8].

The two-staged approach involves waiting for the improvement of the soft-tissue condition, such as the appearance of wrinkles or the epithelialization of blisters, before performing ORIF. However, the soft tissue condition, the waiting period, and the choice of fixation device are left to the surgeon's discretion. In this case, LIFCEF was chosen for the treatment considering the soft-tissue injury and the planned reconstruction of the soft tissue using RSAF. Soft-tissue defect around the ankle joint is challenging for soft-tissue reconstruction due to the limited amount of skin and subcutaneous tissue and the tendency for bones and tendons to be exposed [2]. Skin grafts cannot be used for exposed bones and tendons, and local flaps are unsuitable for reconstruction due to the lack of soft tissues and mobility. Free flaps require microsurgery techniques and a lengthy surgery time. In this case, we performed reconstruction of the soft tissue on the medial side of the ankle joint solely within the orthopedic surgery department using RSAF. RSAF is easy to perform, has a survival rate of 95.2%, and has been reported to be useful for reconstructing soft tissue around the ankle [9].

There have been comparative studies on the use of internal fixation versus external fixation for the definitive fixation of tibial pilon fractures with some reports showing no difference in outcomes between the two methods [10], and others reporting high rates of arthrosis and delayed union with external fixation, but no difference in the incidence of deep infections [11]. However, many of these reports used either mono-lateral or circular external fixation. Circular external fixation is reported to have higher stability from biomechanics and to be more favorable for bone formation with less pin loosening and infection compared with mono-lateral external fixation [12]. Legg et al. conducted a systematic review of definitive fixation using circular external fixation and found an incidence of osteomyelitis and deep infection of 4.8%, demonstrating the usefulness of circular external fixation [13]. In a meta-analysis of ORIF versus LIFCEF, LIFCEF was reported to have a high incidence of arthrosis but no difference in the incidence of deep infection. However, the possibility of bias due to the selection of LIFCEF in cases with more severe articular comminution and greater soft-tissue injury was pointed out [14]. A randomized controlled study of ORIF and LIFCEF showed no difference in treatment outcomes between the two treatments [15]. Based on these reports, there is currently no evidence to indicate the superiority of either ORIF or LIFCEF for the definitive fixation of tibial pilon fractures. However, in cases like the present one that require soft-tissue reconstruction, it is considered useful to use the orthoplastic approach combining LIFCEF with flaps.

## 4. Conclusion

Tibial pilon fractures are difficult to treat due to articular comminution and soft-tissue injuries caused by high-energy trauma. The timing and choice of treatment are crucial concerning the soft tissue. In this case, we treated a closed tibial pilon fracture with soft-tissue necrosis on the medial side with an orthoplastic approach using an RSAF for soft-tissue reconstruction and LIFCEF that obtained a good result.

## Abbreviations

AO/OTA: AO Foundation/Orthopedic Trauma Association
CCS: cannulated cancellous screw
RSAF: Reverse sural artery flap
LIFCEF: Limited internal fixation combined with a circular external fixator
LIFEF: Limited internal fixation combined with an external fixator
ORIF: Open reduction and internal fixation.
